# Fabricating a low-temperature synthesized graphene-cellulose acetate-sodium alginate scaffold for the generation of ovarian cancer spheriod and its drug assessment†

**DOI:** 10.1039/d3na00420a

**Published:** 2023-09-01

**Authors:** Pooja Suryavanshi, Yohaan Kudtarkar, Mangesh Chaudhari, Dhananjay Bodas

**Affiliations:** a Nanobioscience Group, Agharkar Research Institute G. G. Agarkar Road Pune 411 004 India dsbodas@aripune.org; b 2. Savitribai Phule Pune University Ganeshkhind Road Pune 411 007 India; c Department of Mechanical Engineering, Vishwakarma Institute of Technology (VIT) Bibwewadi Pune 411 037 India

## Abstract

3D cell culture can mimic tumor pathophysiology, which reflects cellular morphology and heterogeneity, strongly influencing gene expression, cell behavior, and intracellular signaling. It supports cell–cell and cell–matrix interaction, cell attachment, and proliferation, resulting in rapid and reliable drug screening models. We have generated an ovarian cancer spheroid in interconnected porous scaffolds. The scaffold is fabricated using low-temperature synthesized graphene, cellulose acetate, and sodium alginate. Graphene nanosheets enhance cell proliferation and aggregation, which aids in the formation of cancer spheroids. The spheroids are assessed after day 7 and 14 for the generation of reactive oxygen species (ROS), expression of the hypoxia inducing factor (HIF-1⍺) and vascular endothelial growth factor (VEGF). Production of ROS was observed due to the aggregated tumor mass, and enhanced production of HIF-1⍺ and VEGF results from a lack of oxygen and nutrition. Furthermore, the efficacy of anticancer drug doxorubicin at varying concentrations is assessed on ovarian cancer spheroids by studying the expression of caspase-3/7 at day 7 and 14. The current findings imply that the graphene-cellulose-alginate (GCA) scaffold generates a reliable ovarian cancer spheroid model to test the efficacy of the anticancer drug.

## Abstract 2

3D cell culture can mimic tumor pathophysiology, which reflects cellular morphology and heterogeneity, strongly influencing gene expression, cell behavior, and intracellular signaling.

## Graphical abstract

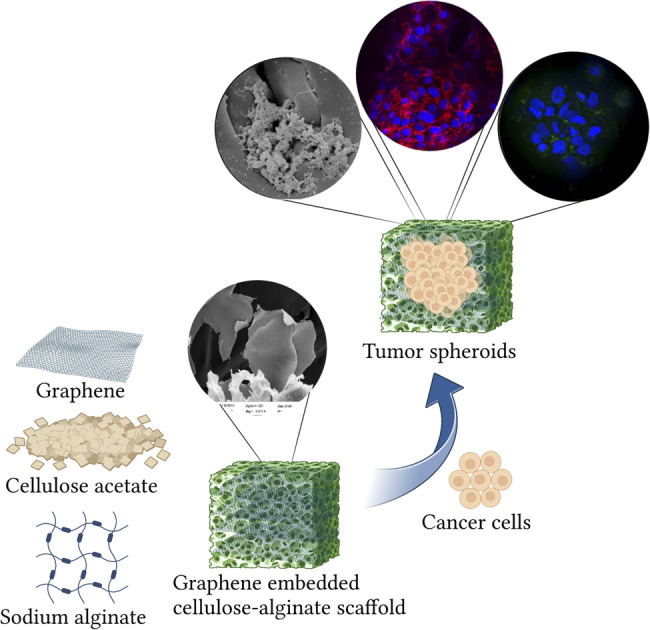

## Introduction

In incidence and diagnosis, ovarian cancer is the eighth most common gynecological cancer worldwide.^1^ Surgery and chemotherapy (carboplatin and taxanes)^2^ are used in its treatment. However, only around 50% of patients benefit from the treatment with adverse side effects,^3^ consequently demanding an effective alternative. Developing new therapeutics is challenging, expensive, and time-consuming, as many drugs fail in clinical trials,^4,5^ due to the lack of *in vitro* models that mimic *in vivo* conditions. Therefore, a reliable primary drug screening model is required to investigate the effectiveness of novel therapies.

Globally, researchers have been employing three-dimensional (3D) cell culture platforms for drug screening as they provide the advantage of cell–cell and cell–matrix interaction. It represents the physiological properties of the cell: cellular morphology and heterogeneity, which strongly influence gene expression, cell behavior, and intracellular signaling.^6,7^

Lately, porous scaffold-based methods to generate tumor spheroids have been used. Porous scaffolds are vital in cell adhesion, proliferation, and migration, as they allow cells to form aggregates and produce the extracellular matrix (ECM).^8^ The interconnectivity of pores and high surface area with macro-and microstructural features influence cell survival, signaling, growth, and motility. The scaffold's mechanical stiffness helps maintain the adhered tumor's phenotype and aggressiveness, influencing the intercellular organization and metastasis ability.^9^ Furthermore, the spheroid mimics the pathophysiology of the *in vivo* tumor and provides a microenvironment to influence its behavior.^10^

Khan *et al.* developed a thiol–acrylate hydrogel scaffold to generate breast cancer spheroids for high throughput drug screening and endocrine response monitoring.^11^ Obayemi *et al.* fabricated microporous paclitaxel loaded PLGA-PEG and PLGA-PCL scaffolds by a particulate leaching process to treat triple-negative breast cancer.^12^ Xu *et al.* demonstrated a chitosan-chondroitin sulphate 3D porous scaffold to promote epithelial to mesenchymal transition in prostate cancer^13^ Yang *et al.* fabricated an alginate-gelatin 3D scaffold to generate cancer spheroids and tumoroids.^14^ Le *et al.* demonstrated the effect of a chitosan–alginate porous scaffold on the proliferation and growth of MDA-MB-231 cells.^15^ Wu *et al.* developed a microfluidic-based vessel-assisted heterotypic tumor model for preclinical drug screening.^16^

Herein, we report the generation of ovarian cancer spheroids in a graphene-cellulose acetate-sodium alginate (GCA) scaffold followed by assessment of drug efficacy. Graphene exhibits unique physicochemical properties such as small size and high surface area, aiding cell attachment and increasing cell growth.^17–19^ The hydrophilic nature of graphene permits interaction with extracellular matrix (ECM) components through hydrogen bonds and electrostatic and covalent interactions, enhancing cell adherence.^20^ Additionally, it is highly adherent to the phospholipid bilayer and polar head groups of the outermost layer of the cell membrane.^21^ Moreover, graphene is carbon-based and less toxic to cells and tissues and, thus, serves as an attractive biocompatible material for tissue engineering and developing regenerative medicine.^22^

Recently, 3D graphene foams have been used as a scaffold for a neural stem cell culture^23^ and differentiation^24^ that supports cell attachment. Chen *et al.* investigated the proliferation and differentiation of induced pluripotent stem cells (iPSCs) on graphene and graphene oxide-based surfaces.^25^ Pilato *et al.* fabricated a graphene oxide-based 3D porous scaffold for cardiac tissue engineering.^26^

Sodium alginate is a naturally derived and biocompatible polymer widely used to culture different types of cells.^27,28^ Furthermore, cellulose acetate is biologically inert and forms nanofibers with surface porosity, promoting cell adhesion, cellular migration, infiltration, tissue ingrowth, and vascularization.^29^

Analytical, morphological, and biological studies were used to characterize high-quality graphene synthesized at low temperature by a modified Hummer's method.^30,31^ The GCA scaffold is fabricated by varying graphene concentrations and assessed for spheroidal structure generation, cell adhesion, viability, and proliferation. Furthermore, the ovarian cancer spheroids generated in the porous GCA scaffold are subjected to doxorubicin. The spheroid is assessed for expression of early apoptotic cell death marker caspase-3/7.

## Results and discussion

Polymer-based 3D scaffolds offer high water-holding capacity due to their porous structures. The flexibility and stiffness of the scaffold support cell–cell and cell–matrix interaction by secretion of the extracellular matrix. Furthermore, it permits the transfer of nutrients and the removal of waste products from tissues without disturbing the culture conditions.^32^ The interconnectivity of the pores in the scaffold aids in the perfusion of nutrients and oxygen, replicating native tissue vasculature.^33^ 3D scaffolds promote the generation of cancer spheroids through cellular aggregates from micrometers to millimeters and offer a better cancer drug screening model than 2D *in vitro* cell culture.^34^

### Cellulose-alginate (CA) scaffolds

Different fabrication methods are used to fabricate the scaffolds, including electrospinning, freeze-drying,^35^ 3D printing,^36^ and drop-casting.^16^ In the present work, cellulose acetate and sodium alginate are drop cast to form a cellulose-alginate (CA) scaffold. The drop-casting technique provides ease of fabrication, aiding in uniformity over the size and thickness of the scaffold.

**Fig. 1:**
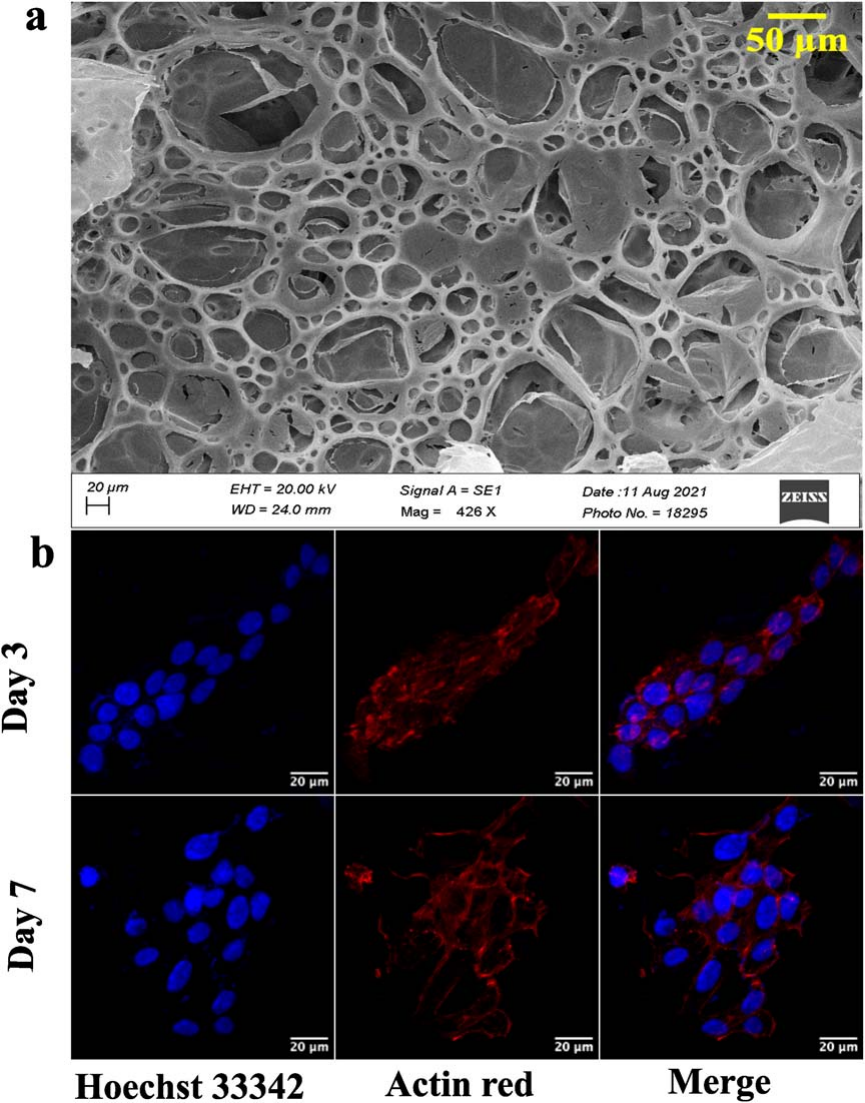
(a) The SEM image of a CA scaffold showing an average pore size 50 μm. (b) Confocal image of the NIH 3T3 cells grown on the CA scaffold on day 3 and day 7. Cell nuclei are stained with Hoechst 33342, and the cytoskeleton is stained with actin red. All the images are captured at a magnification of 63×.

The thickness of the fabricated CA scaffolds is 0.5 mm, and the scanning electron micrograph (Fig. 1a) shows an average pore size of 50 μm. Porosity and tortuosity, which indicate pore distribution and interconnection, are calculated using eqn (1) and (2) as 0.74 and 1.01, respectively.1
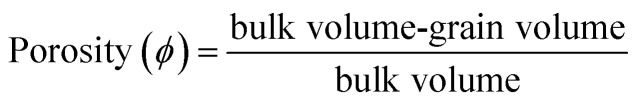
2
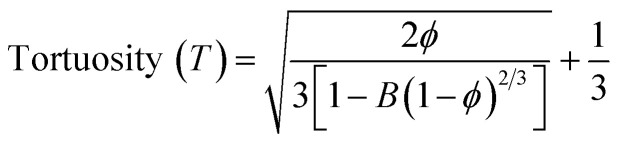
where *B* is calculated as a free parameter with a value of 1.09.^37,38^

Furthermore, NIH 3T3 cells are grown on the scaffold, and their growth is assessed by confocal microscopy after day 3 and 7 (refer to Fig. 1b). An increment in cell growth from day 3 to day 7 is evident. However, the CA scaffold surface did not aid cellular aggregation.

### Characterization of surface morphology, topology, and toxicity of graphene

Graphene is synthesized at low temperature using a modified Hummer's method. Briefly, graphite is reduced to graphitic oxide, followed by a further reduction to reduced graphene oxide (rGO) to obtain high-quality graphene. The product at every synthesis step is characterized structurally and analytically using scanning electron microscopy (SEM), atomic force microscopy (AFM), Raman spectroscopy, Fourier transform infrared (FTIR) spectroscopy, ultraviolet-visible (UV-Vis) spectroscopy, X-ray diffraction (XRD), and X-ray photoelectron spectroscopy (XPS) techniques.

**Fig. 2:**
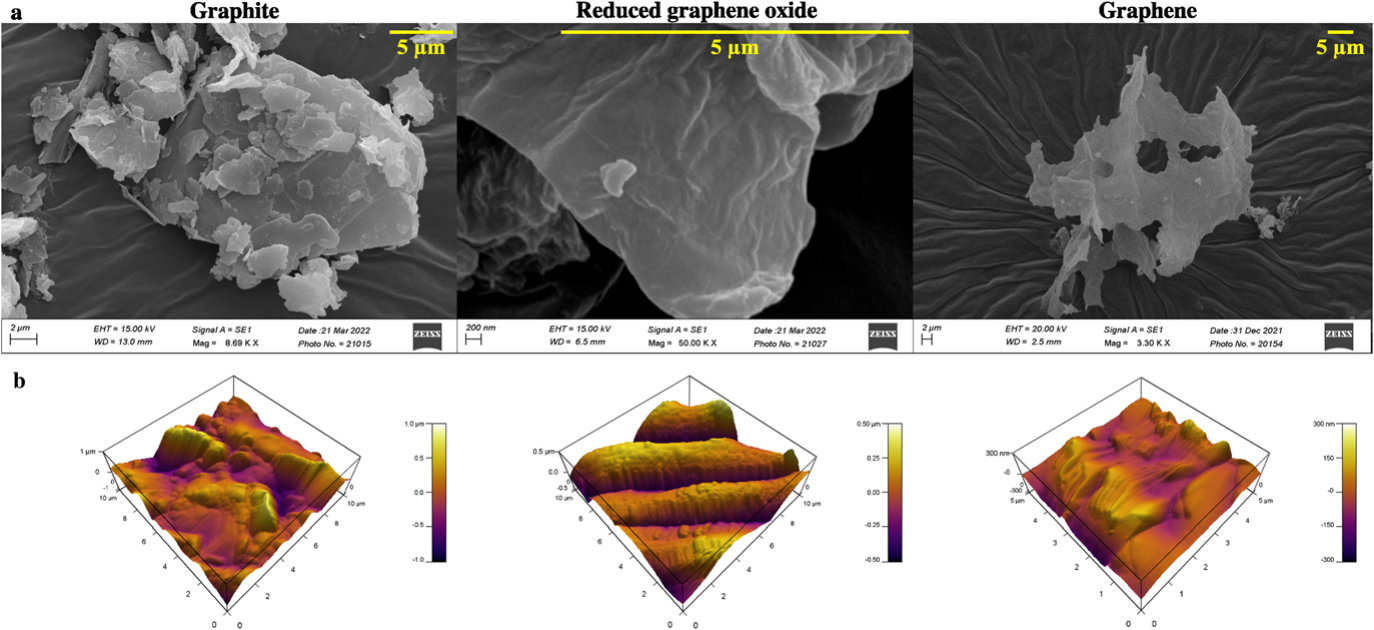
(a) SEM and (b) AFM images for determining the surface morphology and topology of graphite, reduced graphene oxide, and graphene.

Graphite, rGO, and graphene structures are shown in the SEM images (Fig. 2a). Overlapping layers are visible in graphite as thick granules. In contrast, a reduced graphene oxide sample shows non-separated layers (Fig. 2a). The graphene sample, however, has defined layers with distinct edges.

AFM study of the surface topology is conducted to verify the SEM results. There is no evidence of separation between the sheets of carbon in the graphite image obtained using an AFM (Fig. 2b). rGO demonstrates the initiation of sheet separation (refer to Fig. 2b), whereas the graphene sample shows separated sheets, which are noticeably distinct.^39,40^

Preliminary assessments of the precursors graphite, reduced graphene oxide, and graphene are performed using UV-Vis, FTIR, and XRD (refer to Fig. S1†). UV-Vis spectra show a redshift from 256 nm to 231 nm when graphite is converted to reduced graphene oxide. At the same time, a blue shift to 260 nm is associated with high-quality graphene. FTIR analysis didn't show much change as C–C bonds are predominant in all the samples. All the samples show a crystalline nature on characterization using XRD. However, a minor shift is observed for graphene compared to rGO.

**Fig. 3:**
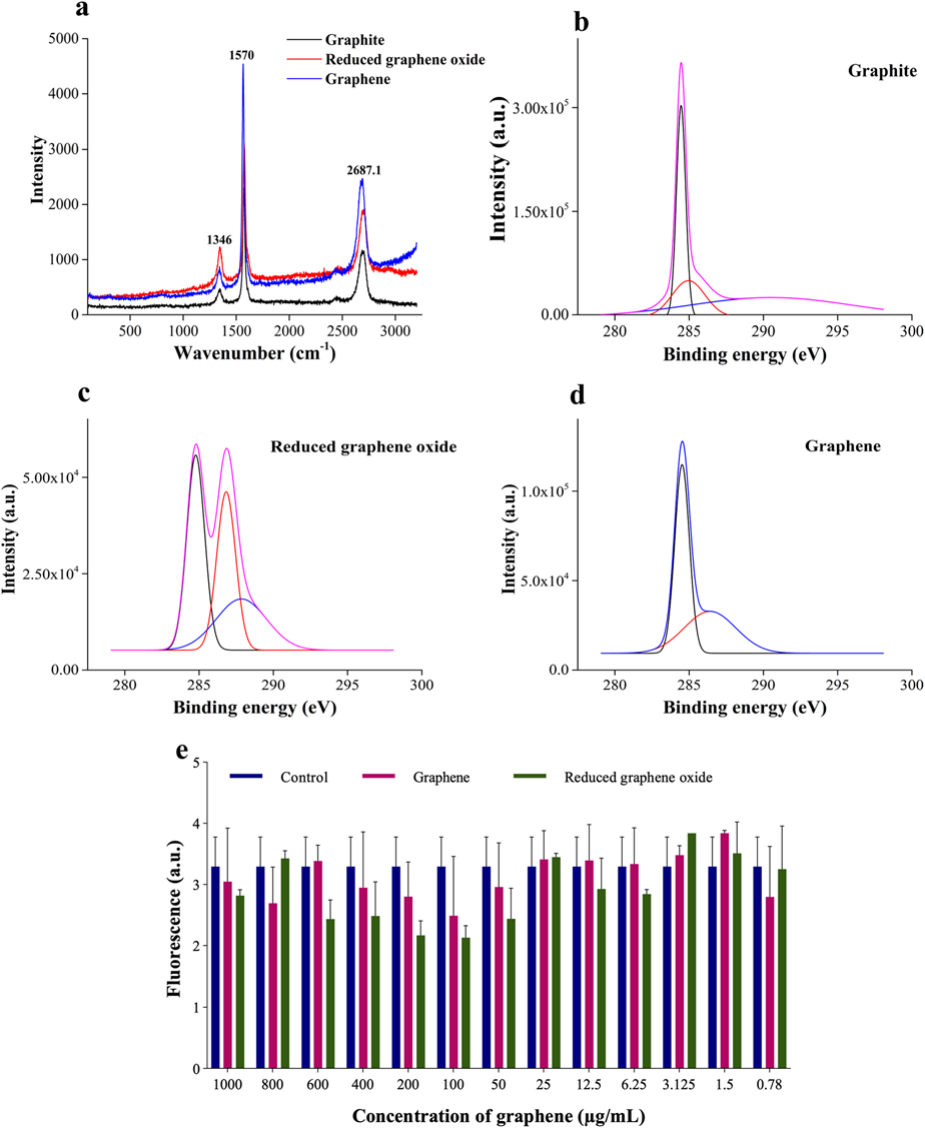
(a) Raman spectra of graphite, reduced graphene oxide, and graphene. X-ray photoelectron spectrograph of (b) graphite, (c) reduced graphene oxide, and (d) graphene, and (e) toxicity assay for graphene and reduced graphene oxide.

Raman spectroscopy and X-ray photoelectron spectroscopy (XPS) are carried out to assess the chemical composition accurately and, to prove the quality of the synthesized graphene. The Raman peak for the 2D band for graphite is observed at 2687.2 cm^−1^, whereas the redshifted one at 2708.1 cm^−1^ is seen for rGO (refer to Fig. 3a). Graphene is obtained by heating rGO at 120 °C, resulting in a blue shift to 2692.8 cm^−1^. The production of high-quality graphene is confirmed by an overall blue shift from graphite to graphene (Fig. 3a). The formation of separated layers in graphene caused by heating is responsible for the blue shift.^41^

Additionally, XPS analysis is performed to evaluate the elemental composition (Fig. 3b). The peak at 285 eV corresponds to the 1s orbital of sp^2^ hybridized carbon. The binding energy of the C–OOR bond is observed at 288.9 eV, while the C–O and C=O bonds are seen from 286–287 eV. The deconvoluted peaks of graphite, reduced graphene oxide, and graphene show varying concentrations of these groups. Graphite shows predominantly sp^2^ hybridized C–C bonds and a shake-up peak at 289.02 eV. The residual oxygen content of rGO is seen to be reduced during the heating process to obtain graphene. Additionally, a sample containing many sp^2^ carbons will have an asymmetric tail in the direction of increased binding energy for the C1s spectrum.^42^ This is clear in the graphene spectrum, which attests to its excellent quality. A similar result is obtained by EDAX analysis (refer to Table S1†). The oxygen percentage reduces by ∼12% after heating the rGO to obtain graphene. Furthermore, an increase in carbon content (∼13%) is recorded, thus proving the purity of synthesized graphene. Furthermore, the graphene is tested for cytotoxicity by 3-(4,5-dimethylthiazol-2-yl)-2,5-diphenyltetrazolium bromide (MTT) assay on the NIH 3T3 (mouse fibroblast) cell line. NIH 3T3 cells are treated for 24 h with different concentrations (1 μg mL^−1^ to 1000 μg mL^−1^) of graphene and rGO. The results (Fig. 3e) show that graphene is non-toxic across all concentrations. The control well has cells without graphene or rGO.

### Graphene-cellulose-alginate (GCA) scaffold

Graphene has drawn considerable attention due to its electrical conductivity, mechanical characteristics, and excellent biocompatibility. Additionally, graphene can boost stem cell differentiation and promote cell adhesion and proliferation. Consequently, graphene scaffolds are employed for developing a 3D cell culture platform.^36^ The 3D cell-supporting structures integrated with graphene offer considerable promise for tissue engineering; for instance, 3D printed graphene scaffolds provide 3D structures with mechanical strength, encouraging *in vivo* bone regeneration by providing physicochemical cues.^43^

**Fig. 4:**
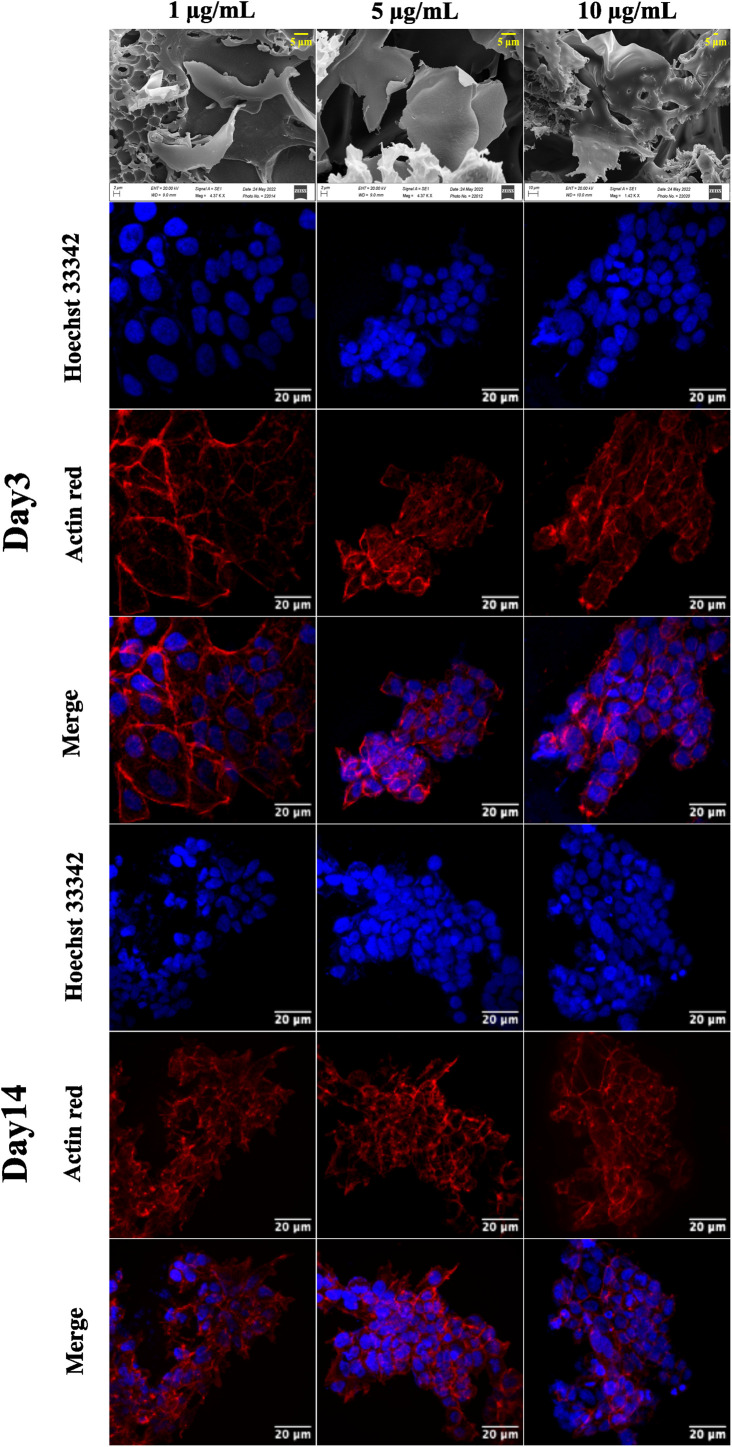
Representative SEM images of the GCA scaffold at different concentrations (1 μg mL^−1^, 5 μg mL^−1^, and 10 μg mL^−1^) of graphene and confocal images of the NIH 3T3 cells grown on the GCA scaffolds captured after day 3 and day 7. The nucleus is stained with Hoechst 33342, and the cytoskeleton is stained with actin red. All the images are captured at a magnification of 63×.

The CA scaffolds are modified with graphene nanosheets to improve cell proliferation and growth. A composite GCA scaffold is fabricated with 1, 5, and 10 μg mL^−1^ graphene concentrations. The SEM of the GCA scaffold (Fig. 4) indicates that graphene sheets are uniformly dispersed throughout the porous scaffold. Furthermore, using the mathematical formulae (eqn (1) and (2)), the porosity and tortuosity of the GCA scaffold are calculated to be 0.91 and 1.21, respectively. Higher porosity and tortuosity values suggest the superiority of the GCA scaffold over the CA scaffold. Additionally, GCA scaffolds outperform CA scaffolds with respect to water retention and absorption capability.

### Cell growth assessment on the GCA scaffold

GCA scaffolds with various graphene concentrations are evaluated qualitatively for cell proliferation, adhesion, and aggregation. Confocal images of NIH 3T3 cells cultured on GCA scaffolds (1 μg mL^−1^, 5 μg mL^−1^, and 10 μg mL^−1^) up to 7 days are shown in Fig. 4.

On day 3 and day 7, the cells are fixed, stained, and imaged. On day 3, many cells are attached to the surface; nonetheless, cellular aggregates are found on day 7 (refer to day 3 and day 7 panels of Fig. 4). Aggregation is enhanced for GCA containing 5 μg mL^−1^ graphene, demonstrating an optimal surface area for spheroid formation.

### Generation of ovarian cancer spheroids on the GCA scaffold

**Fig. 5:**
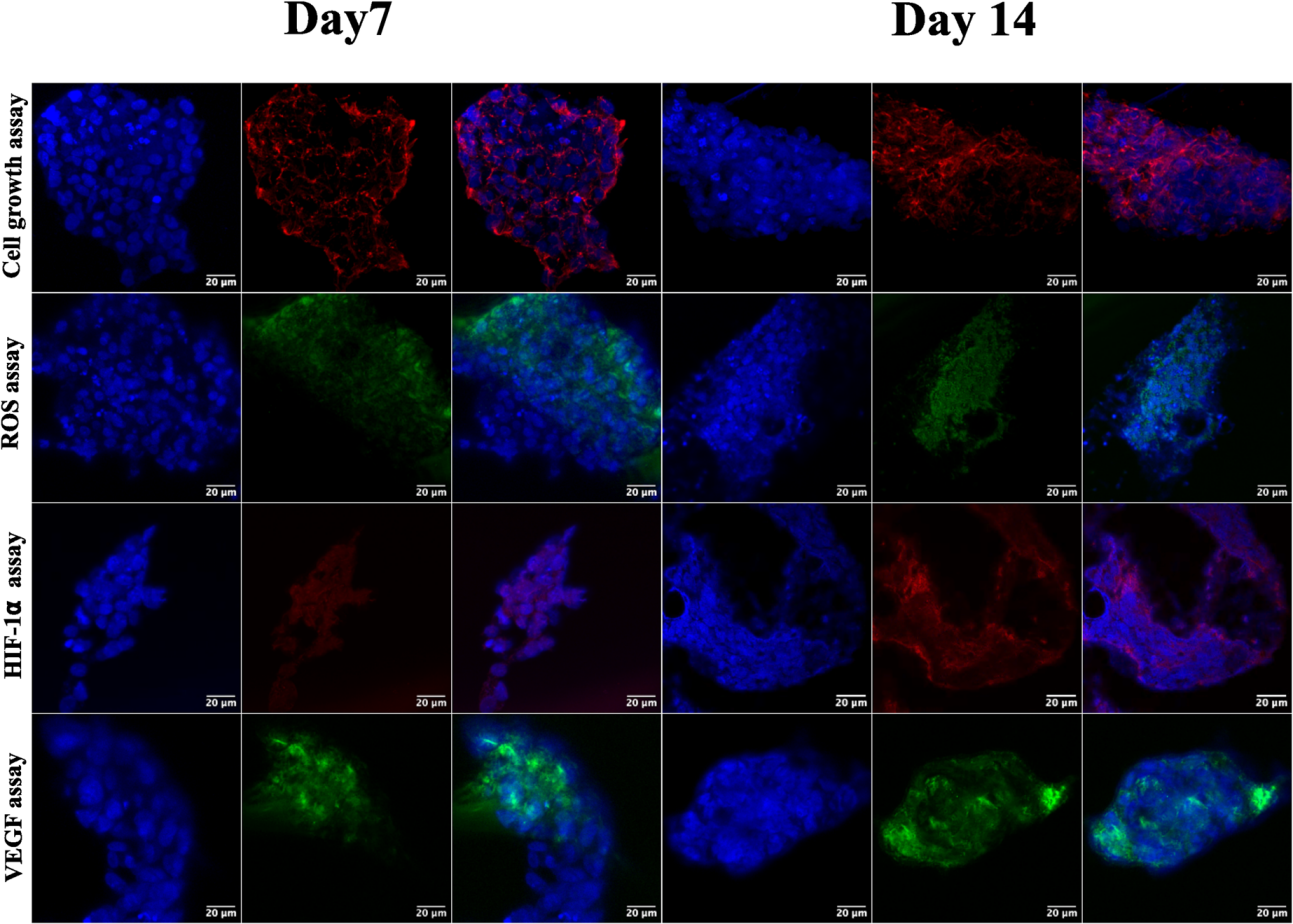
Representative confocal images of ovarian cancer spheroids captured on days 7 and 14 for cell growth assay, formation of ROS, expression of HIF-1⍺, and expression of VEGF. For cell growth assay, the cytoskeleton is stained with actin red. The formed ROS are stained with CellROX green, and expressed HIF-1⍺ is labeled with HIF-1⍺ recombinant rabbit primary monoclonal antibody and Alexa Fluor Plus secondary antibody. The VEGF is labeled with VEGF monoclonal antibody and Alexa Fluor™ 488 secondary antibody. The cell nuclei are stained with Hoechst 33342. All the images are captured at a magnification of 63×.

Following the confirmation of NIH 3T3 cell growth, viability, and cell aggregates, ovarian cancer spheroids are generated using a PA-1 (ovarian teratocarcinoma) cell line. A panel of the cell growth assay (Fig. 5) demonstrates an increment in cell growth from day 7 to day 14, which is also confirmed by scanning electron microscopy (refer to ESI Fig S3†). The formation of reactive oxygen species on day 7 and day 14 is confirmed by staining the spheroids with the CellROX green reagent. Due to the close packing of spheroids, the maximum production of ROS is observed on day 14. Furthermore, the production of the hypoxia inducing factor (HIF-1⍺) and vascular endothelial growth factor (VEGF) confirms the creation of a hypoxic core, which is associated with forming new blood vessels. On day 14, the expression of HIF-1⍺ and VEGF is at its maximum.

**Fig. 6:**
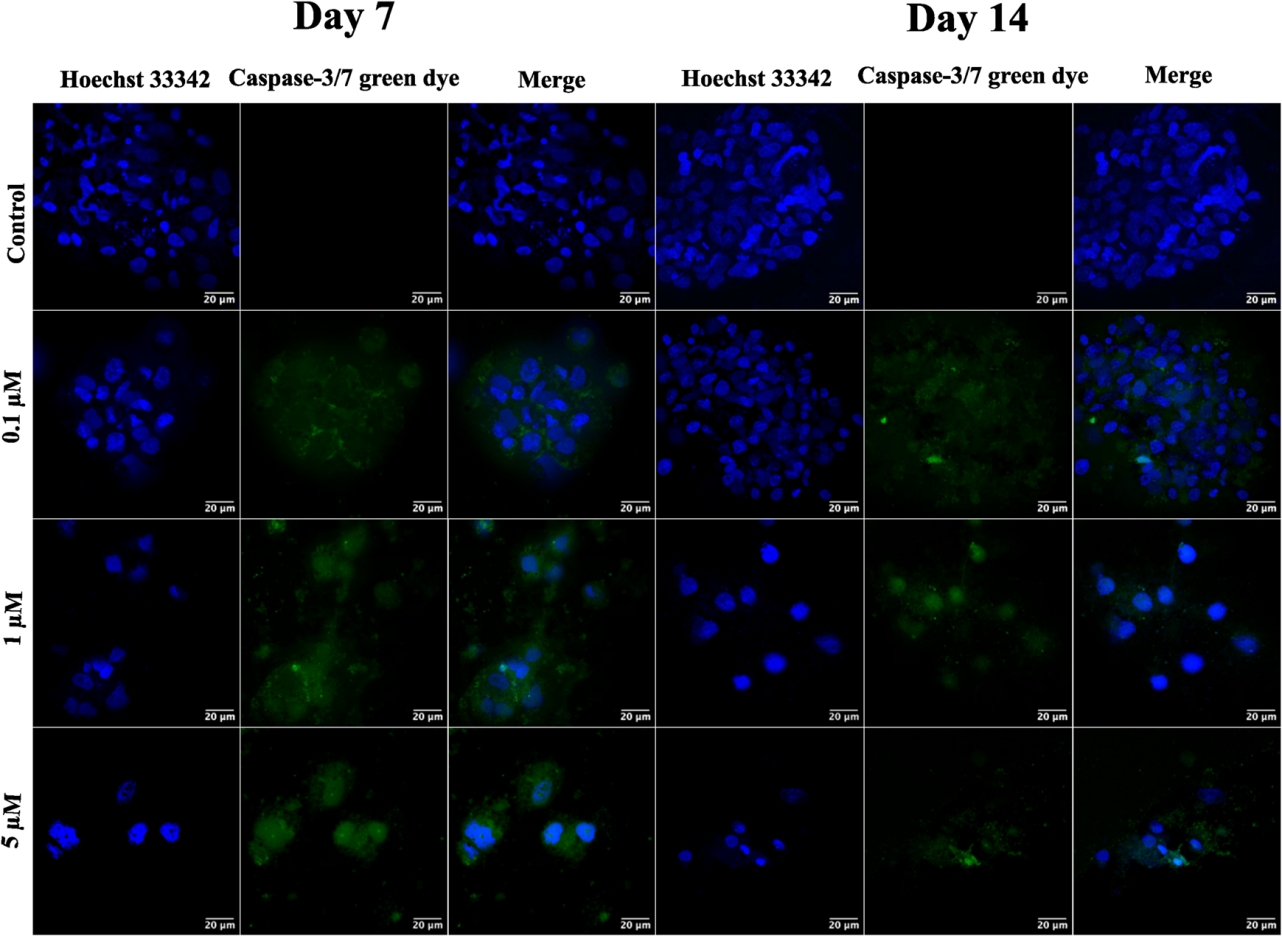
Representative confocal images of ovarian cancer spheroids captured on days 7 and 14 for the expression of caspase-3/7 markers after treatment with different concentrations (0.1 μM, 1 μM, and 5 μM) of doxorubicin. Caspase-3/7 markers are stained with a caspase-3/7 green detection kit, and cell nuclei are stained with Hoechst 33342. All the images are captured at a magnification of 63×.

After validating the tumor spheroids and their characteristic properties, such as reactive oxygen species (ROS) generation and expression of HIF-1⍺ and VEGF, they are treated with doxorubicin at 0.1, 1, and 5 μM.^44^ Spheroids are treated with the caspase-3/7 green detection dye after 24 h to assess the expression of early apoptotic markers. Caspase-3/7 are expressed in ovarian cancer spheroids, along with inhibition of tumor mass and fragmentation of nuclei as can be seen in Fig. 6.

## Discussion

Developing and screening therapeutics using a conventional 2D cell culture platform affects the structural microenvironment resulting in altered cell function and drug response. Therefore, the effective mimicry of *in vivo* physiological conditions could help better translate therapeutics to avoid drug failure in clinical trials. Consequently, 3D scaffolds have been widely used in investigating cancer therapeutics as they increase cell adhesion and proliferation.^45^ In this study, a cellulose acetate and sodium alginate porous scaffold is cast, characterized, and assessed for cell adherence and growth on NIH 3T3 cells. Even though the scaffold is porous with interconnected pores (refer to Fig. 1a), confocal images (Fig. 1b) indicate the absence of cell aggregates on the scaffold. The scaffold should provide a large surface area for cells to aggregate and form a spheroidal structure. Thus, graphene nanosheets are added to the CA scaffold as it is reported to aid in adhesion and enhance cell growth, resulting in the spheroidal development of cancer cells.^17,46^

Graphene nanosheets should possess the requisite characteristics to aid in the formation of spheroidal structures. Moreover, the hydrophilic surface of graphene nanosheets ensures interaction with the ECM through hydrogen bonds and electrostatic and covalent interaction, enhancing cell adherence. Thus, graphene is synthesized from graphite at low temperature (120 °C) using a modified Hummer's method.^31^ A literature review reveals that graphene is synthesized at over 180 °C. However, low temperature will be advantageous in the synthesis of longer nanosheets, resulting in a larger surface area. The morphological characterization reveals the precise separation of graphene layers as seen in SEM images (Fig. 2a).^30,31^ AFM images confirm the edge definition and distinct layers of graphene, indicating its quality (Fig. 2b).^30^

Furthermore, the XRD spectra show peak-broadening caused by varied sizes of graphene sheets (refer to ESI Fig. S1c†).^30,31^ The well-defined nanosheets and higher degree of freedom are responsible for the smooth peaks obtained from the FTIR spectrum (refer to ESI Fig. S1b†). This demonstrates that the van der Waals interaction forces are overcome, resulting in independent graphene nanosheets.^30^ A sharp XRD peak is observed in graphite because the non-separated layers of carbon are in the same 〈*hkl*〉 plane (refer ESI Fig. S1c†). However, the XRD of graphene shows peak broadening because of the unordered stacking and separation of layers.

A correction in the peak of the 2D band in Raman spectra (Fig. 3a) reveals the separation of graphene sheets.^31^ Furthermore, a blue shift in the graphene peak is suggestive of the quality of the synthesized graphene.^30^

The elemental analysis of graphene carried out by EDAX shows increased carbon content and a decreased oxygen concentration (refer to ESI Table S1†). The deconvoluted XPS peaks of graphite, reduced graphene oxide, and graphene show the varying concentrations of C–O, C=O, and C–OO bonds. Graphite primarily has sp^2^ hybridized C–C bonds and entrapped oxygen between the layers. The carbon–oxygen bonds are broken due to chemical reduction, increasing the peak intensity of the sp^2^ carbon for rGO.^31^ The heating of rGO released the entrapped oxygen resulting in high-purity graphene. Thus, well-defined randomly oriented graphene sheets are obtained and visible from SEM and AFM micrographs, indicating their good quality.

The cytotoxicity assay (Fig. 3e) demonstrates that graphene inhibits cell growth at a higher concentration ranging from 600 to 1000 μg mL^−1^ and increases cell growth at lower concentrations than the control.^47^ The drop-cast graphene (1 μg mL^−1^, 5 μg mL^−1^, and 10 μg mL^−1^) cellulose acetate sodium alginate (GCA) scaffolds show porosity and tortuosity of 0.91 and 1.21 respectively. The higher values, compared to the CA scaffold, indicate an increase in surface area and interconnection of pores. Furthermore, GCA scaffolds with 5 μg mL^−1^ graphene show increased cell growth with cellular aggregates for NIH 3T3 cells, indicating spheroidal formation (Fig. 4).

The results obtained from cell viability data (refer to ESI Fig. S2†) show a significant increment in cell viability at the same graphene concentration. 1 μg mL^−1^ of graphene does not provide sufficient surface area for cell adherence due to the low concentration of graphene. In contrast, 10 μg mL^−1^ graphene shows stacking of graphene layers hindering the cell adherence on the scaffold (Fig. 4a). The GCA scaffold is hydrophilic and possesses a larger surface area due to the presence of nanosheets, resulting in increased cell adhesion and proliferation. As the extracellular matrix is not used, cells cluster, communicate with one another and secrete their own ECM to form cancer spheroids.^48^

The ovarian cancer spheroids generated on the 5 μg mL^−1^ GCA scaffold show increased tumor mass from day 7 to day 14. ROS are generated from day 7 to 14 (Fig. 6) from the cancer spheroids,^49^ indicating the aggregation onset with increased ECM secretion. Cell growth in a confined space creates stress in the cells due to the scarcity of oxygen, nutrients, space constraints, and acidic pH. Furthermore, the absence of gaseous exchange creates hypoxic conditions in the core of cancer spheroids, which triggers the expression of HIF-1⍺ followed by the VEGF.

Finally, the formed tumor spheroids are treated with doxorubicin, a chemotherapeutic drug, at varying concentrations. After 24 h of treatment, a reduction in tumor mass with distorted cell morphology and fragmentation of cell nuclei is observed. However, complete tumor killing after 24 h of drug treatment is not observed due to the creation of drug resistance in the cancer spheroids. In contrast, complete killing is observed in 2D cell culture, where cells are exposed to the drug that quickly penetrates due to monolayer structures and unnatural conditions.^50^

## Experimental section

### Fabrication of cellulose-alginate (CA) scaffolds

Scaffolds are fabricated by mixing 1% sodium alginate M.W. 749 g mol^−1^ (Sigma Aldrich USA), 1% calcium chloride (HiMedia), and 10% cellulose acetate with average molecular weight 100 000 (Acros Organics) in a ratio of 5 : 1 : 10 followed by drop casting 1 mL of the mixture on a cover slip. The scaffolds are allowed to polymerize overnight at room temperature.

### Synthesis of graphene

2 g graphite (Sisco Research Laboratory (SRL) and 2 g NaNO_3_ (HiMedia) are added to 90 mL H_2_SO_4_ (98%) (SDFCL) and stirred for 30 min in an ice bath. 10 g KMnO_4_ (Qualigens) is added slowly and stirred at 50 °C for 2 h, then 200 mL deionised (DI) water and 12 mL H_2_O_2_ (Sigma Aldrich USA) are gradually added until the solution cools to room temperature. The solution is centrifuged at 5000 rpm for 60 min, and the supernatant is decanted. The product is washed with DI water and centrifuged repeatedly until the pH turns neutral (7.0). Thus, the graphitic oxide is dissolved in DI water and ultrasonicated for 1 h to yield graphene oxide. The suspension is centrifuged at 5000 rpm for 60 min, and the supernatant is decanted. Graphene oxide is mixed with DI water, followed by NaBH4 (LOBA CHEMIE) addition, and further reduced with 10% HCl (HiMedia). The reduced graphene oxide (rGO) is washed with DI water and purified by repeated centrifugation for 60 min at 5000 rpm, followed by lyophilization to obtain a powder. The rGO powder is heated until flashpoint (120 °C) to obtain pure graphene.

### Fabrication of a graphene-cellulose-alginate (GCA) scaffold

Graphene powder obtained by a modified Hummer's method at low temperature is dissolved in DI water to create different concentrations (1, 5, and 10 μg mL^−1^). It is added to the CA scaffold to obtain the GCA scaffold.

### Analytical, morphological, and toxicological characterization

The fabricated scaffolds are characterized for their morphology and pore distribution using a scanning electron microscope (SEM) EVO MA15, Zeiss (Germany). An atomic force microscope (AFM) from Nanonics, Israel, is used to analyze the surface of graphene. Raman spectra are obtained using a Renishaw Raman spectrometer, and an ESCA-3000 XPS is used to assess the purity of graphene. An X-ray diffractogram (XRD) is recorded to analyze the crystallinity, and Fourier transform infrared spectroscopy (FTIR) is used for bonding. UV-Vis spectroscopic data is assessed to deduce the interaction of graphite, rGO, and graphene with light.

Graphene is assessed for cytotoxicity by toxicity assay (MTT) using NIH 3T3 (mouse fibroblast cells). 1 × 10^4^ cells per well are seeded in 96 well plates and allowed to grow for 24 h, followed by treatment with 1 μg mL^−1^ to 1000 μg mL^−1^ graphene. After treatment, 2 mg mL^−1^ of 3-(4,5-dimethylthiazol-2-yl)-2,5-diphenyltetrazolium bromide (MTT) (Thermofisher Scientific) is added to each well and incubated at 37 °C for 4 h. 200 μL of DMSO is added and absorbance is recorded at 512 nm using a plate reader.

### 3D cell culture on CA and GCA scaffolds

NIH 3T3 cells (purchased from the National Centre for Cell Science (NCCS) repository, Pune, India) are grown in Dulbecco's modified Eagle’s medium (DMEM), 10% fetal bovine serum (FBS), and 1% antibiotic antimycotic (Invitrogen USA). CA and GCA scaffolds are washed with 70% ethanol, followed by washing with double distilled water twice and autoclaved. Furthermore, 1 × 10^6^ NIH 3T3 cells are seeded on scaffolds and allowed to grow. On days 3 and 7, scaffolds are washed with phosphate buffered saline (PBS), fixed with 4% paraformaldehyde, and permeabilized with 0.5% Triton X-100 (Sigma Aldrich USA). Cell nuclei are stained with Hoechst 33342, and the cytoskeleton with rhodamine-phalloidin (Invitrogen USA) and visualized under a confocal microscope (Model TCS SP8, Leica, Germany).

To generate ovarian cancer spheroids, PA-1 (ovarian teratocarcinoma) cells (purchased from the National Centre for Cell Science (NCCS) repository, Pune) are maintained in minimum essential medium (MEM) with 10% FBS and 1% antibiotic antimycotic (Invitrogen USA). 1 × 10^6^ PA-1 cells are seeded on the scaffolds, and the growth of ovarian cancer spheroids is assessed. The ovarian cancer spheroids are observed for the generation of reactive oxygen species (ROS) and expression of the hypoxia-inducing factor (HIF-1⍺) and vascular endothelial growth factor (VEGF) on days 7 and 14. The formation of ROS is checked by labeling the spheroids with a CellROX green reagent (Invitrogen USA). Furthermore, the expression of HIF-1⍺ is checked by fixing the spheroids with 4% paraformaldehyde and permeabilized with 0.5% Triton-X-100 (Sigma Aldrich USA). The cells are blocked with 2% bovine serum albumin (BSA) (Sigma Aldrich, USA), followed by treatment with HIF-1⍺ recombinant rabbit monoclonal primary antibody (Invitrogen, USA) in PBST (1× PBS, 0.5% BSA and 0.02% Tween-20), and labeled with donkey anti-rabbit IgG (H + L) highly cross-adsorbed secondary antibody, Alexa Fluor™ Plus (Invitrogen USA). Similarly, the expression of VEGF is checked by labeling VEGF monoclonal antibody (Invitrogen USA) and rabbit anti-mouse IgG (H + L) cross-adsorbed secondary antibody, Alexa Fluor™ 488 (Invitrogen USA).

### Effect of doxorubicin on ovarian cancer spheroids

Spheroids of PA-1 grown on GCA scaffolds are tested for efficacy of the anticancer drug doxorubicin after day 7 and 14. The spheroids are treated with 0.1, 1, and 5 μM doxorubicin hydrochloride (European Pharmacopoeia Reference standard) for 24 h.^51^ A control group is maintained under similar experimental conditions as compared to the test group; however, the control group is not treated with drugs. The expression of the early apoptotic marker is checked by staining the spheroids with a CellEvent™ caspase-3/7 green detection kit (Invitrogen USA) and observed under the confocal microscope.

## Conclusions

This paper reports the fabrication of a GCA scaffold for the growth of ovarian cancer spheroids. A low-temperature modified Hummer's synthesis method yields high-quality and non-toxic graphene nanosheets. The GCA scaffold shows a porosity and tortuosity of 0.91 and 1.21, respectively, indicating its porous nature with interconnected pores. Due to a higher surface area, enhanced animal cell growth and adhesion are obtained using graphene nanosheets. Furthermore, graphene plays a crucial role in forming cellular aggregates. The spheroid formation closely resembles the tumor morphology and characteristic features of the *in vivo* tumor microenvironment by expressing ROS, HIF-1⍺, and VEGF. The ovarian cancer spheroids are subjected to different concentrations of doxorubicin and assessed after days 7 and 14. The drug treatment results show the expression of caspase-3/7, an early apoptotic marker, indicating the apoptotic nature of cell death. Thus, the GCA scaffold is an excellent substrate for cellular adhesion, proliferation, spheroid formation, and drug screening. Further developments can lead to the generation of cancer spheroids with tumor properties and serve as an alternative tool to rapid and high throughput drug screening platforms. Adding perfusion and microfluidics to the spheroid model will enable a close mimicry of *in vivo* conditions.

## Author contributions

Dhananjay Bodas conceptualized, designed, and supervised the work and contributed to data analysis. Pooja Suryavanshi fabricated and characterized the graphene-cellulose-alginate (GCA) scaffold, generated ovarian cancer spheroids, characterized the structures, and performed studies with the drug. Yohaan Kudtarkar synthesized graphene and characterized the product using morphological and analytical techniques. Mangesh Chaudhari was involved with the graphene characterization. The manuscript was written and revised with the contributions of all authors. All authors have approved the final version of the manuscript.

## Conflicts of interest

The author declares no conflict of interest.

## Supplementary Material

NA-005-D3NA00420A-s001
