# Supplementary material for: Fabricating a low-temperature synthesized graphene-cellulose acetate-sodium alginate scaffold for the generation of ovarian cancer spheriod and its drug assessment
Source: Nanoscale Adv. 2023 Sep 1;5(18):5045–53. doi: 10.1039/d3na00420a (PMC10496900; doi:10.1039/d3na00420a)
Supplement: NA-005-D3NA00420A-s001 [file NA-005-D3NA00420A-s001.pdf]

Electronic Supplementary Material (ESI) for Nanoscale Advances.
This journal is © The Royal Society of Chemistry 2023

Fabricating low-temperature synthesized graphene-cellulose acetate-sodium alginate scaffold for the generation and drug assessment of ovarian cancer spheroids

Pooja Suryavanshi,[ab] Yohaan Kudtarkar,[c#] Mangesh Chaudhari,[c] and Dhananjay Bodas*[ab]

Characterization of graphene

UV-Vis Spectrophotometer (UV-400) and IR Affinity-1 Fourier Transform Infrared Spectrophotometer (Shimadzu) are used to analyze the characteristics of the material. FTIR spectra are scanned from 400-4000 cm^{-1}. Rigaku XRD was used to analyze the crystallographic pattern of the material. Further, with the integration of the Oxford EDS system, impurities and the quantity of carbon were analyzed. Moreover, GCA scaffolds are evaluated for cell viability using a resazurin-based Prestoblue® reagent. The cell viability is measured by seeding 1×10^6 NIH 3T3 cells on the scaffold embedded with varying concentrations of graphene (1, 5, and 10 µg/mL) and assessed after 1, 3, and 7 days. Cells are trypsinized from the scaffold, and presto blue reagent was added in a 10:100 ratio and incubated for 30 min. The fluorescence intensity was recorded at 530/590 nm on days 1, 3, and 7.

UV-Vis Spectroscopy

The absorption peak at 256 nm in the UV–Vis spectrum of graphite corresponds to $\pi - \pi^*$ transitions of the remaining *sp*^{2} C=C bonds, which were shifted to a shorter wavelength (231 nm) after the reduction of graphite to reduced graphene oxide (rGO). A further shift to a longer wavelength is observed from 231 nm of rGO to 260 nm for graphene (Fig.S1a) after heating till the flashpoint. Heating rGO to graphene increased the π-conjugation, i.e., restored the π-conjugation network (as in graphite case), resulting in a lower energy requirement for the transition.

Fourier Transform Infrared spectroscopy.

The FTIR spectra show a reduction in bound (3450 cm^{-1}) and unbound (3640 cm^{-1}) -OH, C-O, and C=O with the heating step. The graphene ranges have a similar pattern with reduced noise compared with the graphite spectra. The noise reduction is because graphite, composed of multiple layers, has few degrees of freedom, whereas graphene possesses separate layers. As all three samples are forms of carbon, the signature peaks can be seen from all the spectra visibly seen in (Fig.S1b). However, there is very little difference between graphite and graphene when considering the type of bonds observed.

X-ray Diffraction Analysis

XRD spectra prove the benefit of heating to obtain graphene. The graphitic carbon shows a sharp peak at 26.35°. The reduced graphene oxide shows the peak at 26.54° which shifts to 25.52° after the final heating step. Peak broadening is observed in graphene due to the unordered stacking of sheets. This stacking of sheets is because of the high static interaction between the layers. The slight shift between graphite and graphene suggests the presence of some residual oxygen content in the produced graphene (Fig.S1c).

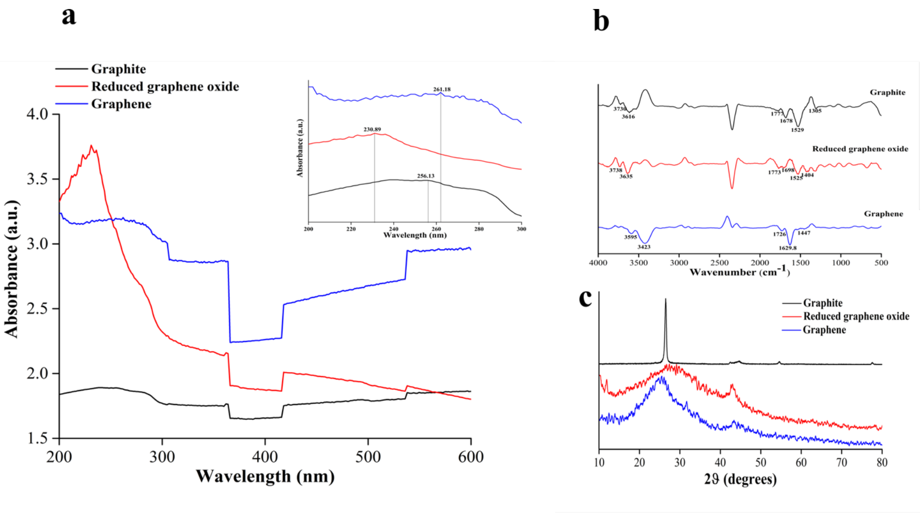

Figure S1 a) UV-Vis spectra, b) FTIR spectra, and c) XRD spectra of graphite, reduced graphene oxide, and graphene.

Energy dispersive X-ray Analysis

The heating step leads to an increase in the concentration of carbon in the obtained graphene, thereby decreasing oxygen content and other impurities. A monolayer of oxygen is formed under high vacuum conditions; thus, oxygen contamination cannot be avoided. Consequently, few oxygen molecules are present in the graphene and cannot be removed with the help of chemical techniques. The sample must be heated at high temperatures in an inert atmosphere to reduce the oxygen impurities. Some sodium is present in the selection and is constant across all regions. Its presence might be due to sodium nitrate and sodium borohydride. Depth profiling checks the purity of different graphene layers and avoids the substrate effect. At low acceleration voltage, the presence of silicon substrate is negligible.

Table S1: Variation in the elemental concentrations of graphite and the synthesized rGO and graphene.

Elements	Atomic (%)		
	Graphite	rGO	Graphene
C	91.55	60.02	73.75
O	8.45	34.64	22.92

Cell viability

A significant increment in cell viability from day 1 to day 7 on the scaffold containing 5 µg/mL graphene concentration was observed, which can be seen in Fig.S2.

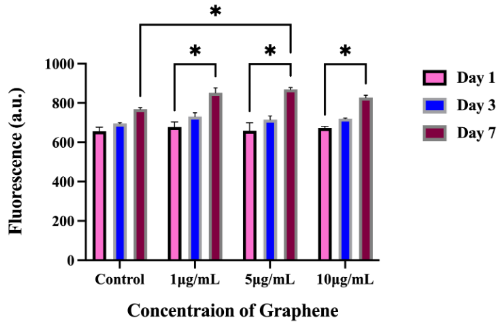

Fig S2. Graph representing the growth of NIH 3T3 cells on the GCA scaffold with varying concentrations of graphene (1, 5, and 10 µg/mL) on days 1, 3, and 7.

Scanning Electron Microscopy

The generated ovarian cancer spheroids on GCA scaffolds are confirmed by scanning electron microscopy on days 7 and 14. On days 7 and 14, cells are washed with PBS and fixed with 0.2% glutaraldehyde solution (Sigma Aldrich USA) at 4°C for 12 h. The glutaraldehyde solution is discarded, and scaffolds are submerged in 10% ethanol for 15 min. Further, 10% ethanol is discarded, and the previous step is repeated with 30%, 50%, 70%, 90%, and 100% ethanol. Scaffolds containing cancer spheroids are dried in a critical point drier, coated with gold, and observed under a scanning electron microscope.

Increased tumor mass and aggregation are observed from days 7 to 14, as shown in Fig S3.

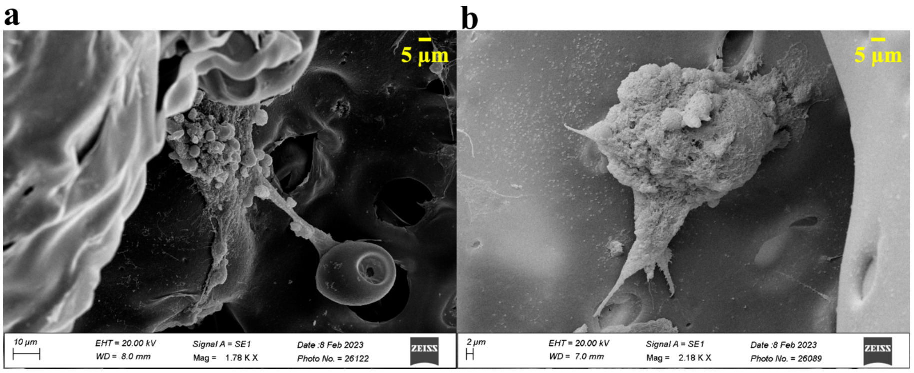

Fig S3 SEM images of ovarian cancer spheroids grown on GCA scaffold on a) day 7 and b) day 14.
